# Evaluation of canine detection of COVID‐19 infected individuals under controlled settings

**DOI:** 10.1111/tbed.14529

**Published:** 2022-04-05

**Authors:** Anne‐Lise Chaber, Susan Hazel, Brett Matthews, Alexander Withers, Guillaume Alvergnat, Dominique Grandjean, Charles Caraguel

**Affiliations:** ^1^ School of Animal and Veterinary Sciences University of Adelaide Roseworthy South Australia Australia; ^2^ School of Animal and Veterinary Sciences University of Adelaide Roseworthy South Australia Australia; ^3^ Detector Dog Program, Operational Strategy and Coordination Australian Border Force Bulla Australia; ^4^ Department Special Operations Metropolitan Fire Service South Australia Adelaide Australia; ^5^ International Affairs Bureau Ministry of Interior of the UAE Dubai United Arab Emirates; ^6^ Ecole Nationale Vétérinaire d'Alfort Université Paris Est Maisons‐Alfort France

**Keywords:** COVID‐19, detection dogs, diagnostic accuracy, diagnostic sensitivity, diagnostic specificity, SARS‐Co‐2 canine detection, screening tool

## Abstract

Reverse transcription polymerase chain reaction (RT‐PCR) is currently the standard diagnostic method to detect symptomatic and asymptomatic individuals infected with Severe acute respiratory syndrome coronavirus 2 (SARS‐CoV‐2). However, RT‐PCR results are not immediate and may falsely be negative before an infected individual sheds viral particles in the upper airways where swabs are collected. Infected individuals emit volatile organic compounds in their breath and sweat that are detectable by trained dogs. Here, we evaluate the diagnostic accuracy of dog detection against SARS‐CoV‐2 infection. Fifteen dogs previously trained at two centres in Australia were presented to axillary sweat specimens collected from known SARS‐CoV‐2 human cases (*n* = 100) and non‐cases (*n* = 414). The true infection status of the cases and non‐cases were confirmed based on RT‐PCR results as well as clinical presentation. Across dogs, the overall diagnostic sensitivity (DSe) was 95.3% (95%CI: 93.1–97.6%) and diagnostic specificity (DSp) was 97.1% (95%CI: 90.7–100.0%). The DSp decreased significantly when non‐case specimens were collected over 1 min rather than 20 min (*p* value = .004). The location of evaluation did not impact the detection performances. The accuracy of detection varied across dogs and experienced dogs revealed a marginally better DSp (*p* value = .016). The potential and limitations of this alternative detection tool are discussed.

## 1 INTRODUCTION

Severe acute respiratory syndrome coronavirus 2 (SARS‐CoV‐2), first reported in humans in Wuhan, China in December 2019, is the cause of COVID‐19 disease (Wiersinga et al., 2020). As of the 22 September 2021, World Health Organisation (WHO) reported over 228.8 million COVID‐19 confirmed cases and 4.7 million associated deaths globally (WHO Coronavirus Disease (COVID‐19) Dashboard, n.d.). In humans, viral replication and shedding in the upper respiratory tract begins in the pre‐symptomatic phase, 2–3 days prior to the onset of COVID‐19 symptoms (He et al., 2020). Disease modelling from Singapore and China suggested that 48–62% of transmissions come from pre‐symptomatic individuals (Ganyani et al., 2020). People exposed to a positive case or who are pre‐symptomatic may take time to self‐present for testing, leading to disease transmission and a potential outbreak.

To effectively reduce transmission of SARS‐CoV‐2, reliable, scalable, accurate and inexpensive testing to detect both symptomatic and asymptomatic individuals is required (Wiersinga et al., 2020). The standard diagnostic test for COVID‐19 is SARS‐CoV‐2 reverse transcription polymerase chain reaction (RT‐PCR) performed on respiratory specimens (nasopharyngeal swabs or lower respiratory tract samples) (Wiersinga et al., 2020). The detectability varies with the adequacy of specimen collection, time from onset of symptoms and specimen source (Sethuraman et al., 2020; Wang et al., 2020). Performing SARS‐CoV‐2 RT‐PCR testing is labour intensive, time consuming, expensive and susceptible to a shortage of reagents. Cost can be prohibitive for resource poor countries which may also be unable to access reagents. Protracted turn‐around time can hamper case and contact identification, adversely affecting public health responses. A scalable, cost‐effective, non‐invasive, rapid screening tool could improve targeted testing and public health responses, helping to control the spread of disease.

Volatile organic compounds (VOCs) are emitted by our body, breath and sweat, and reflect our metabolic condition (Shirasu & Touhara, 2011). Development of infectious or metabolic disease results in changes in VOCs profile with some being disease specific and potentially used as diagnostic olfactory markers (Shirasu & Touhara, 2011). Canines can detect VOCs and, if formally trained, may be able to discriminate between infected and non‐infected humans in some specific diseases (Guest et al., 2019; McCulloch et al., 2006; Taylor et al., 2018). Recent pilot studies demonstrated that dogs were able to detect patients infected (symptomatic or not) with SARS‐CoV‐2 using respiratory secretion specimens (Jendrny et al., 2020), heat‐treated urine and saliva samples (Essler et al., 2021) and sweat samples (Grandjean et al., 2020). Respiratory secretions are likely to contain viral particles therefore sweat specimens were investigated instead to reduce the risk of infection to the operators (Fathizadeh et al., 2020).

This study evaluated the accuracy of detector dogs in identifying SARS‐CoV‐2‐infected individuals from axillary sweat specimens at two dog training centres in Australia. This report complies with the STARD 2015 standards to report diagnostic accuracy (Cohen et al., 2016).

## 2 MATERIAL AND METHODS

### 2.1 Detection dogs and their trainers

A total of 15 dogs, seven with no experience, six with experience in the detection of explosive compounds from the Australian Border Force (ABF), and two repurposed dogs from the South‐Australian Metropolitan Fire Services (SAMFS) were recruited. ABF dogs were bred, developed and selected for their environmental stability, play and hunt drive. Each dog was consistently handled by the same experienced handler from ABF, SAMFS or the Australian Department of Agriculture.

### 2.2 Specimens sourcing

Specimens were collected from 12 primary and tertiary healthcare facilities (hospitals, clinics, screening stations, etc.) across France, the United Arab Emirates (UAE) and Australia. Only volunteers who were at least 18 years old, provided written consent and agreed to comply with the collection instructions were recruited. Individuals who had recovered from COVID‐19 within the preceding 45 days or received a therapy against SARS‐CoV‐2 infection in the preceding 24 h were not sampled. Axillary (armpit) sweat specimens were collected following the same standard protocol and using the same media across all locations, except for the duration of impregnation. Participants placed one piece of standard sized (7.5 × 7.5 cm) sterile gauze under each armpit in direct contact with the skin for 20 min in France and Australia or for 1 min in the UAE. Impregnated gauzes were transferred into a labelled plastic bag/container. The outer bags/containers were then disinfected with an alcohol wipe and placed into a second bag labelled identically using a no‐touch technique outside the room where the collection occurred. Study staff assisting the collection of specimens from confirmed SARS‐CoV‐2 cases used personal protective equipment (face mask shield, gloves) and procedures in line with WHO and their respective countries’ regulations.

Specimens were shipped refrigerated (+4 to +8℃) to the dog training facilities in Australia by mail and in separate packaging for cases and non‐case specimens. In Australia, specimens were kept refrigerated between testing or frozen for longer term storage. Specimens were not used for longer than 15 days after first bag/container opening.

### 2.3 Specimens’ case definition

A ‘case specimen’ was an axillary sweat sample collected from a participant who yielded a positive RT‐PCR against SARS‐CoV‐2 within 7 days prior collection. A RT‐PCR was considered positive if the cycle threshold value (Ct) was <34, regardless of COVID‐19 symptoms, or if Ct ≤ 40 when the person was (i) symptomatic (anosmia, ageusia, muscle aches, respiratory symptoms, diarrhoea, fever, fatigue, headache) or (ii) with an image scanner and/or clinical picture suggestive of SARS‐CoV‐2 infection or (iii) had a history of recent contact (≤48 h) with a known SARS‐CoV‐2‐infected person. No Ct information was available for tests conducted in the UAE; therefore, any positive RT‐PCR provided by local health authorities was considered positive.

A ‘non‐case specimen’ was an axillary sweat sample collected from a participant who yielded a negative RT‐PCR against SARS‐CoV‐2 (quantification cycle (Cq) > 40) on the day of collection (specimens sourced in France and the UAE) or from a participant who resided in an area with negligible risk of infection (i.e., from a state with no case of SARS‐CoV‐2 community transmission for more than 30 days) and did not experience COVID‐19 symptoms. Specimens from persons without COVID‐19 symptoms and without suspicious history of contact but yielding a Cq value of ≥34 and <40 were not included. All samples were collected on similar swab types (Australian negative specimens were sampled using French or Australian gauzes).

### 2.4 Specimen screening

For detection, specimens were transferred into a clean glass jar and connected to a presentation stainless steel cone (hide) of a construction similar to those developed previously (Grandjean et al., 2020). The study dogs were trained to display a ‘conditioned response’ behaviour (sustained sit with focus on the target) on a hide containing the target odour through reward‐based training techniques. A key training requirement was hide screening independent of handler cues to eliminate the potential ‘Clever Hans bias’. Dogs were trained at two separate sites, in Adelaide (Roseworthy Veterinary School) and Melbourne (ABF Canine Detection Unit), and the same locations were used to assess their detection accuracy. The study dogs were trained following the Standard Operating Protocols provided in Supplementary Material S1.

Within an evaluation run, a total of nine hides were used with one case specimen or none per run. Dog handlers were blinded to both – the hide and the run true status. The presence of a case specimen and the specimens’ hide order was formally randomized using a smartphone application (Random Number Generator ©2013 Nicholas Dean). Running of individual dogs was ordered in such a manner that each dog had an equitable number of first passes on a set of specimens. All specimens used for this study were new to the dogs (not used during training) and were collected from new patients or volunteers sourced from the same locations as for the specimens used during training. Case specimens were used once per dog but could be used with other dogs. Non‐case specimens were used up to eight times within and between dogs (specimens collected from each armpit were used two to four times). Where possible, case and non‐case specimens sourced from the same location were presented in the same run to avoid possible interference of background odours. This was the case for all runs using specimens from UAE. As all but one specimen from France were cases and all specimens from Australia were non‐cases (gauzes from France were also used to collect sweat from Australian non‐cases), these two locations were used conjointly within runs.

A primary data recorder, who was not blinded to the true status of the hides, was located in a booth with one‐way screens so they could have direct sight on the hides but could not be seen by the blinded handler or the blinded secondary/back‐up data recorder. The primary data recorder who had knowledge of the sample status was behind a one‐way screen, always remained silent and could not give any cues during the evaluations. The dog's handler signalled his response to the data recorder by hand gesture. The data recorder then pressed a light visible to the handler to indicate if the dog correctly identified a case sample, allowing the handler to give appropriate positive reinforcement. Reinforcement for positive identifications was used to reduce problems with motivation in the dogs during the extending testing required for these trials.

Data recording involved recording individual specimen identifiers and hide order, whether or not a hide was searched, a dog's search behaviours and the presence (or absence) of any conditioned response (i.e., sitting in front of the hide). Each run was recorded on video for data quality control and assurance. The data from both recorders were then compared at the end of each day and video evidence was examined to resolve any conflicts.

### 2.5 Evaluation of detection accuracy

The evaluation of detection accuracy was conducted at the individual hide level. Hides not sampled by the dog (dog did not screen the hide) were excluded from the analysis.

The detection accuracy was measured using the conventional parameters used for diagnostic test accuracy – diagnostic sensitivity (DSe) and specificity (DSp). Here, the DSe refers to the proportion of hides containing a case specimen where the dog displayed a conditioned response behaviour (i.e., true positive rate), whereas DSp corresponds to the proportion of hides containing a non‐case specimen where the dog did not display a conditioned response behaviour (i.e., true‐negative rate). The DSe complements the rate of false negatives, whereas the DSp reflects the rate of false positives. The DSe and DSp were estimated for (i) 1‐min specimen only (coming from the UAE), (ii) for 20‐min specimen (coming from France and Australia) and (iii) for all specimens combined.

Two separate logistic regression models were built – one for DSe using the results from hides containing only a case specimen and one for DSp using the results from hides containing a non‐case specimen. To estimate the overall DSp across all dogs, the models included ‘dog’ and ‘specimen’ as crossed random effects to account for the fact that a given specimen could be repeatedly screened within and across dogs. For the DSe modelling, only the specimen random effect was used because case specimens were only used once per dog. The effects of dog experience (yes or no) and evaluation location (Adelaide vs. Melbourne) on DSe and DSp were also investigated by including these factors as fixed effect in the models. Comparison of accuracy between dog experience levels and evaluation locations were not investigated for 1‐min specimens (sourced in the UAE) due to the limited number of runs for this source. The population averaged estimates for the models and their 95% CI were reported.

Dog‐specific estimates were only estimated with specimens from France and Australia. Dog was included as a fixed effect within the model with specimen remaining as a random effect. Due to the model estimation approach, dogs with perfect scores were dropped from the model. For those ‘perfect’ dogs, we estimated their DSe or DSp and their corresponding Exact Binomial 95% CI directly and ignoring the repeated usage of specimens. Dog‐specific estimates for 1‐min specimens were not obtained because of the paucity of data.

## 3 RESULTS

### 3.1 Evaluation runs description

A total of 514 impregnated specimens were used during the evaluation runs – 100 were from infected cases (16 from UAE and 84 from France) and 414 were from non‐cases (29 from UAE, one from France and 384 from Australia). Detection results were collected on 931 fully blinded runs completed by 15 dogs over 33 open days across two locations (Adelaide and Melbourne) between the 4 January and the 4 March 2021. Of the completed runs, 86.3% (*n* = 803) included one hide with a case specimen. Each dog completed 54 runs on average (range: 43–67). The final dataset included a total of 5260 hide screenings – 803 with case specimens (703 using French and 100 using UAE specimens) and 4457 with non‐case specimens (12 using French, 3741 using Australian and 336 using UAE specimens).

### 3.2 Overall detection accuracy

Of the 803 hides with a case specimen, 769 yielded a conditioned response from the detection dog. After accounting for same case specimens being used multiple times (i.e., observation not fully independent) across dogs, the overall detection dog DSe was 95.3% (95%CI: 93.1–97.6%). In other words, 4.7% of the case hides are expected to yield a false negative. Of the 4457 hides with a non‐case specimen, 4384 did not yield a conditioned response and, after observation dependence adjustment, the overall diagnostic DSp was 97.1% (95%CI: 90.7–100.0%). That is, 2.9% of the non‐case hides are expected to yield a false positive.

The DSp and, to a lesser extent, the DSe seemed affected by the length of swab placement in the axillary area with 1‐min specimens being detected with less accuracy (Table 1). While the DSp significantly decreased with 1‐min samples (*p* value = .004), the decrease in DSe was not significant (*p* value = .330). The location of the evaluation (Melbourne or Adelaide) and thus between group of dogs involved in this study did not significantly impact the DSe (*p* value = .555) or the DSp (*p* value = .126).

**TABLE 1:** Diagnostic sensitivity (DSe) and specificity (DSe) estimates by length of swab placement in the axillary area

Length of swab placement in the axillary area	Run completed	Case hide count	DSe (95%CI)	Non‐case hide count	DSp (95%CI)
20‐min specimens	814	703	95.8% (93.5%–98.0%)	4,121	98.1% (96.8%–99.5%)
1‐min specimens	117	100	92.6% (85.5%–99.8%)	336	93.6% (88.5%–98.7%)
All specimens	931	803	95.3% (93.1%–97.6%)	4,457	97.1% (90.7%–100.0%)

### 3.3 Dog‐specific detection accuracy

**FIGURE 1:**
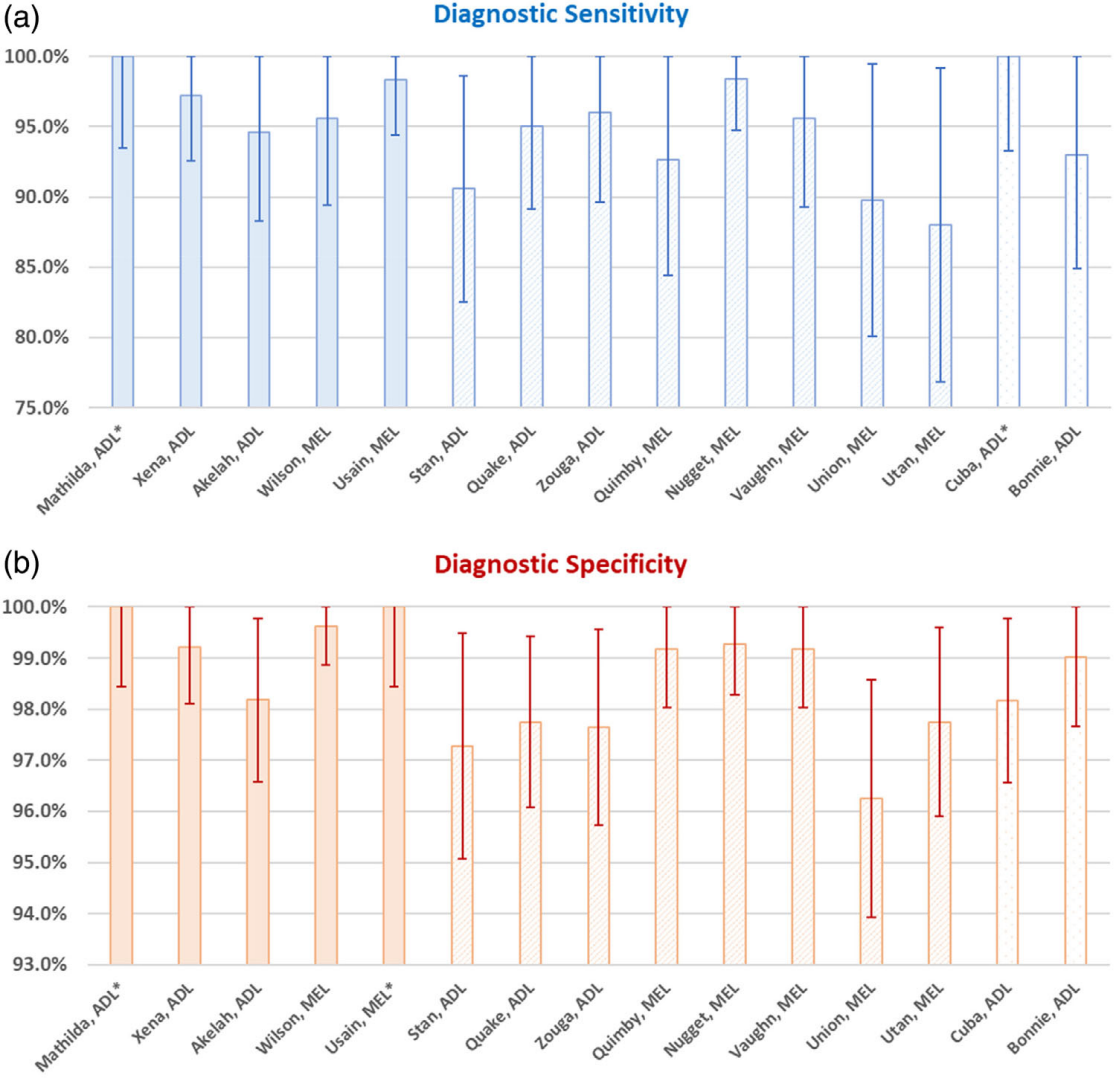
Comparison of diagnostic sensitivity (a) and specificity (b) estimates across individual dogs. The error bars represent the 95% CI of the estimates. Full bars are experienced dogs, dashed bars are inexperienced dogs and dotted bars are repurposed dogs. Be aware of the different *y*‐axis scale used between graphs

Accuracy of detection varied across dogs (Table 2 and Figure 1). Across dogs, the DSe ranged from 88.0 to 100.0% and the DSp from 96.3 to 100.0%. One experienced dog (Matilda) achieved perfect DSe and DSp, one repurposed dog (Cuba) achieved perfect DSe and another experienced dog (Usain) achieved perfect DSp. Experienced dogs did not show a significantly better DSe (*p* value = .222) but showed a significantly better DSp (*p* value = .016) which improved by 1.6% and is therefore only marginally relevant.

**TABLE 2:** Dog‐specific and overall estimates of diagnostic sensitivity (DSe) and specificity (DSp) excluding 1‐min specimens

Dog	Breed	Sex	Age (years)	Evaluation location	Dog detection experience level	Run completed	Case hide screening count	DSe (95%CI)	Non‐case hide screening count	DSp (95%CI)
Mathilda	Labrador	F	8	Adelaide	Experienced	61	55	100.0%^a^ (93.5%–100%)	236	100.0%^a^ (98.4%–100%)
Xena	Labrador	F	3	Adelaide	Experienced	64	56	97.2% (92.5%–100%)	281	99.2% (98.1%–100%)
Akelah	Labrador	F	8	Adelaide	Experienced	67	57	94.6% (88.3%–100%)	323	98.2% (96.6%–99.8%)
Stan	Labrador	M	1	Adelaide	Unexperienced	61	54	90.6% (82.5%–98.6%)	289	97.3% (95.1%–99.5%)
Quake	Labrador	M	1	Adelaide	Unexperienced	66	54	95.0% (89.2%–100%)	381	97.8% (96.1%–99.4%)
Zouga	Labrador	M	2	Adelaide	Unexperienced	53	43	96.0% (89.6%–100%)	273	97.6% (95.7%–99.6%)
Cuba	Labrador	F	4	Adelaide	Repurposed	59	53	100.0%^a^ (93.3%–100%)	315	98.2% (96.6%–99.8%)
Bonnie	Spaniel	F	2	Adelaide	Repurposed	50	43	93.0% (84.9%–100%)	216	99.0% (97.7%–100%)
Wilson	Labrador	M	2	Melbourne	Experienced	48	41	95.6% (89.4%–100%)	242	99.6% (98.9%–100%)
Usain	Labrador	M	2	Melbourne	Experienced	51	42	98.3% (94.4%–100%)	280	100.0%^a^ (98.4%–100%)
Quimby	Labrador	M	1	Melbourne	Unexperienced	43	39	92.6% (84.4%–100%)	248	99.2% (98%–100%)
Nugget	Labrador	M	2	Melbourne	Unexperienced	51	46	98.4% (94.8%–100%)	286	99.3% (98.3%–100%)
Vaughn	Labrador	M	1	Melbourne	Unexperienced	48	42	95.6% (89.3%–100%)	248	99.2% (98%–100%)
Union	Labrador	M	1	Melbourne	Unexperienced	47	40	89.8% (80.1%–99.5%)	258	96.3% (93.9%–98.6%)
Utan	Labrador	M	1	Melbourne	Unexperienced	45	38	88.0% (76.8%–99.2%)	245	97.8% (95.9%–99.6%)

^a^ Because of perfect scores, these estimates could not be modelled and were estimated separately with their Binomial Exact 95% CI.

## 4 DISCUSSION

This study provides evidence to support that detector dogs are an accurate and effective tool to determine people infected with SARS‐CoV‐2 using an easily implemented collection method in placing a gauze swab in the axillary area for a short time. The DSe and DSp of the individual dogs involved in the trial varied, with some operating at 100%, and all comparing favourably with the diagnostic accuracy of RT‐PCR testing.

All results from the detector dogs are compared with RT‐PCR, which are not perfect and whose accuracy depends on viral load being shed. Viral load and thus PCR Cycle quantification (Cq – number of cycles of amplification of the sample genetic material) varies through infection. When the viral load is low (very early stage of infection or during recovery), the Cycle threshold is high; when viral load is high (peak of infection), the Cycle threshold is lower. However, there is no standard on the ‘Cq’ between different laboratories and countries and thus PCR positive or negative results need to be interpreted cautiously. A high Cq poses a risk of patients being misclassified as being actively infected. To reduce the risk of presenting specimens that are falsely considered positive, high Cq values (>34) were paired with clinical information or recent history of contact with COVID‐19 infected persons. However, it was not done for 1‐min specimens (UAE samples) where Cq values were not available, and this can explain the lower DSe and DSp for these specimen origins.

Careful selection of case specimens is both a strength and a limitation of this study. Dogs detect VOCs produced during active infection, but it is unknown for how long the VOCs are emitted by the organism after the infection ceased. It is therefore unknown if dogs can still detect patients that are recovering. Although RT‐PCR on nasopharyngeal swabs will remain positive days after active infection ceased (residual viral RNA genome fragments but no active viral particles), dogs might not be able to detect convalescing patients. Further research using longitudinal swabbing of people infected with SARS‐CoV‐2 is needed to determine the period of infection during which a dog will accurately detect an infection.

The Arevalo‐Rodriguez et al. systematic review reveals that up to 54% of COVID‐19 patients may have an initial false‐negative RT‐PCR. These findings reinforce the need to develop tools able to identify infected patients during the incubation phase. In our study, all dogs gave a conditioned response on two non‐case specimens from the UAE. Retrospective investigations suggested that one patient was potentially in incubation phase during sweat collection as this individual developed symptom (headache, muscle ache, dry cough) shortly after sweat samples’ collection. No laboratory results were available to confirm infection status. The other individual remained asymptomatic.

The UAE specimens were included to trial if dogs could detect VOCs in sweat specimens collected for only 1 min. Although the DSp decreased significantly in the UAE compared with the French samples, dogs were exposed to UAE samples with no initial training to 1‐min samples which might have led to detection threshold issues and can explain the lower DSe on those samples. This study suggests that length of swab placement in the axillary area might affect detector dogs’ diagnostic accuracy. Further research is needed to confirm this finding.

Dogs were trained using case specimens from both the UAE and France, and non‐case specimens from three countries (UAE, France and Australia) from 12 different locations including hospitals and screening stations. This is likely to have helped the dogs to generalize their target scent, as samples originated from different environments, and participants were from different ethnic groups. Detector dogs trained for odours such as explosives and drugs, might have problems generalizing from the odours they are trained on to the more variable odours in the field (Moser et al., 2019). Although the dogs worked in a controlled environment in the current study, the variability of samples makes it more likely they would maintain diagnostic accuracy if deployed to a new environment. On the other hand, sourcing most non‐cases from the same country could help dog's discrimination. To reduce this issue, we have diversified the source of cases and we have used gauzes from the same sources to collect non‐case specimens in Australia. Sampling protocols were identical in all countries and transit time was similar with shipment of specimens lasting 2–3 days from overseas to Australia and 2 days within Australia. Case and non‐case specimens were collected in the same locations in the UAE and dogs’ results suggest that discrimination remains accurate in these conditions.

SARS‐CoV‐2 evolves through time and undergoes mutations and recombination which might alter the VOCs profile. Although no genomic data were available from case specimens sampled in this project, it is extremely likely that dogs were exposed to several SARS‐CoV‐2 variants during training and validation exercises due to the timeline of this study and the diverse source of samples (France and the UAE). Genomic data of the variants circulating in the world were accessed on nextstrain.org on a weekly basis during the life of this project and highlighted the evolution and diversity of SARS‐CoV‐2 variants from the source populations. The dogs trained in this study were able to generalize to new strains with no further training. For ongoing use of this new disease screening tool, we recommend that their olfactory memory library is regularly updated by exposing them to recent cases from a variety of variants and strains.

Dogs were trained using positive reinforcement‐based methods, with food and/or a toy used as the reward. Positive reinforcement has been shown to be the most effective method of dog training, it ensures the dog welfare while building a positive relationship with the dog handler (Ziv, 2017). There is a lack of scientific studies of specific training protocols for odour detection by dogs in order to determine which are the most effective in terms of time to train to criterion and accuracy of detection (Hayes et al., 2018). In a study using rats trained to detect odours, an intermixed training method (including more than one odour at a time) was more effective than sequential single‐odour training (Keep et al., 2021). In the present study, the sweat samples would have presented an intermixed odour, which may have helped in training the dogs to generalize across different samples. With the potential use of detector dogs for not only COVID but also other diseases such as malaria (Guest et al., 2019), further research is needed to optimize the selection and training methods used with these dogs.

Although the dogs may generalize the scent of a case for SARS‐CoV‐2 infection, context is also important for detector dogs (Gazit et al., 2005). In the early stages of deployment and in a new environment, it will be important to validate their sensitivity and specificity again prior to full deployment. An important facet of the training protocol used is that an axillary sweat sample can be easily and quickly provided by people. There is also the potential for dogs to screen people directly, for example, dogs could scent the axillary area of people while they are seated. Other protocols using respiratory or heat inactivated urine or saliva samples (Essler et al., 2021; Jendrny et al., 2020) may not be amenable to deployment in areas such as airports due to either risk of infection or inability to supply the sample in a timely manner.

Although this study has verified the diagnostic accuracy of detector dogs for SARS‐CoV‐2 infection in humans, it is important to understand the limitations of extrapolating from a controlled to an operational setting. It is impossible to replicate an operational setting in a controlled study. For example, dogs working in the field on a daily basis may encounter zero prevalence of SARS‐CoV‐2 positive samples or clusters with much higher prevalence than tested in this study. It will be important to verify the diagnostic accuracy of detector dogs for SARS‐CoV‐2 human infections in future studies as these dogs are deployed.

## 5 CONCLUSION

This study demonstrates the diagnostic accuracy of detector dogs for screening people infected with SARS‐CoV‐2. Detector dogs may not replace the existing screening with RT‐PCR, but could be a complementary method that could be quickly and effectively deployed to provide immediate results. Their additional value may lie in being able to detect infection in pre‐symptomatic people before the virus is shed and when RT‐PCR is still negative. Further research is needed to uncover which VOC is specific to SARS‐CoV‐2 infection and to assess VOC persistence through the course of infection. Our study shows that trained dogs can accurately detect SARS‐CoV‐2 infection using axillary sweat samples. Canine screening has potential as a scalable, rapid, efficient and reliable tool.

## CONFLICT OF INTEREST

The authors declare that there is no conflict of interest.

## ETHICAL STATEMENT

The study protocol was approved by the Animal ethics committee of the University of Adelaide (approval reference number: S‐2020‐052), by the United Arab Emirates Ministry of Health and Prevention Research Ethics Committee (approval reference number MOHAP/DXB‐REC/SSS/No 131/2020) and by the Protection of Persons Committee (CPP) of the Ile‐de‐France. This research is part of the study ‘COVIDEF’ promoted by Assistance Publique ‐ Hopitaux de Paris (Parisian hospitals, AP‐HP), as ‘COVIDOG’ (Cohort of patients infected by the virus SARS‐CoV‐2 or suspected to be infected), for the samples coming from AP‐HP, and of the study « VOC‐COVID‐Diag » promoted by Foch hospital (Suresnes). The latter study has been approved by the CPP Nord Ouest IV (approval reference number: ID‐RCB 2020‐A02682‐37).

## Supporting information

Supporting Information

## Data Availability

The data that support the findings of this study are available on request from the corresponding author. The data are not publicly available due to privacy or ethical restrictions.
