# Supplementary material for: Evaluation of canine detection of COVID‐19 infected individuals under controlled settings
Source: Transbound Emerg Dis. 2022 Apr 5:10.1111/tbed.14529. Online ahead of print. doi: 10.1111/tbed.14529 (PMC9115492; doi:10.1111/tbed.14529)
Supplement: Supplementary file 1 — Supporting Information [file TBED-9999-0-s001.docx]

**COVID-19 Detector Dog Project**


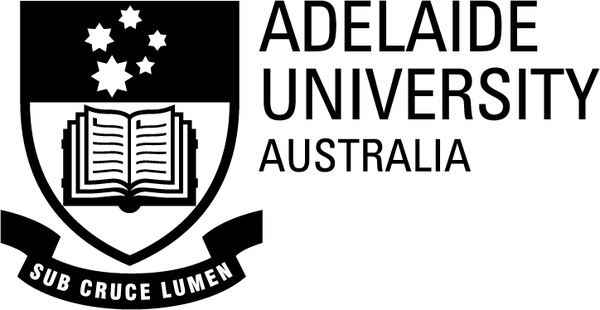


**Procedures for the training and validation of detector dogs**

**for the**

**International COVID-19 Detector Dog Study**

Contents

[**Acknowledgements:** 2](#_Toc54036910)

[**Purpose** 4](#_Toc54036911)

[**Scope and application** 4](#_Toc54036912)

[**Alert behaviour and scent training chambers (hides)** 4](#_Toc54036913)

[**Section 1 – Fundamental Skills Development** 6](#_Toc54036914)

[**1.1** **Establishing final response alert behaviours** 6](#_Toc54036915)

[**1.2** **Introduction to a training odour** 8](#_Toc54036916)

[**1.3** **Introduction to scent discrimination** 9](#_Toc54036917)

[**1.4** **Introduction to search** 10](#_Toc54036918)

[**1.5** **Introduction to a blank search** 11](#_Toc54036919)

[**1.6** **Validation of fundamental skills development and / or project acclimatisation** 11](#_Toc54036920)

[**Section 2: Introduction to target odour (all dogs)** 13](#_Toc54036921)

[**Section 3: Training Validation Test** 18](#_Toc54036922)

[**Section 4: Guides for delivery of training sessions** 19](#_Toc54036923)

[**Training Record Sheet** 21](file:///C:\Users\withersa\Desktop\Covid%20DD\COVID%20DD%20SOP_v4.docx#_Toc54036924)

# **Acknowledgements:**

The following sources were reviewed and referenced in the development of this document

- Prof Dominique Grandjean, Prof Raid Sarkis, Mme Clothhilde Lecoq-Julien: May 20^th^, 2020, NOSAIS – Video Conference Presentation - NOASIS COVID 19 Program.
- Prof Dominique et al 2020, Detection dogs as a help in the detection of COVID-19: Can dogs alert on COVID-19 positive persons by sniffing axillary sweat samples? – Proof of concept study. bioRxiv reprint doi: ***https//doi.org/10.1101/2020.06.03.132134***

This document was developed by Senior Fire Fighter Alex Withers, Metropolitan Fire Service South Australia, Project Officer (South Australian Study Group) COVID-19 Detector Dog Project, University of Adelaide.

#

# **Purpose**

The purpose of this document is to develop a standardised set of guidelines for the training, development and validation of dogs participating in the International COVID Detector Dog study under the University of Adelaide.

The training and development of canines during this phase of the project will involve 3 key stages;

1. **Fundamental Skills Development** – (‘Green’ dogs and / or trained search / detector dogs sourced from allied disciplines not involving passive alert behaviours).
2. **Imprint to Target Odour** – (requires acclimation to study hides for dogs with prior training as detector dogs)
3. **Training Validation Test**

The objective is to develop a core group of detector dogs that on completion of a Training Validation Test will be able to participate in a proof of concept trial consistent with the International study (French and UAE studies).

On completion of the training dogs will also undertake continuation training in accordance with proposed deployment models currently being scoped in consultation with international project partners.

# **Scope and application**

Dogs will be trained to detect the signature of volatile organic compounds (VOC) in the sweat of COVID 19 positive patients and be able to discriminate between COVID positive and negative samples.

Samples will be obtained from the sweat from the arm pits of both COVID positive and negative subjects (donors). The samples will be contained within chambers (hides) of a construction similar to those used in the French and UAE proof of concept studies.

Line ups of up to 10 hides will be arranged and the canine asked to systematically search each hide for the presence of a COVID positive sample.

On successful completion of the Training Validation Test the dog and handler team will be recommended for participation in the double blind ‘Proof of Concept’ trail.

# **Alert behaviour and scent training chambers (hides)**


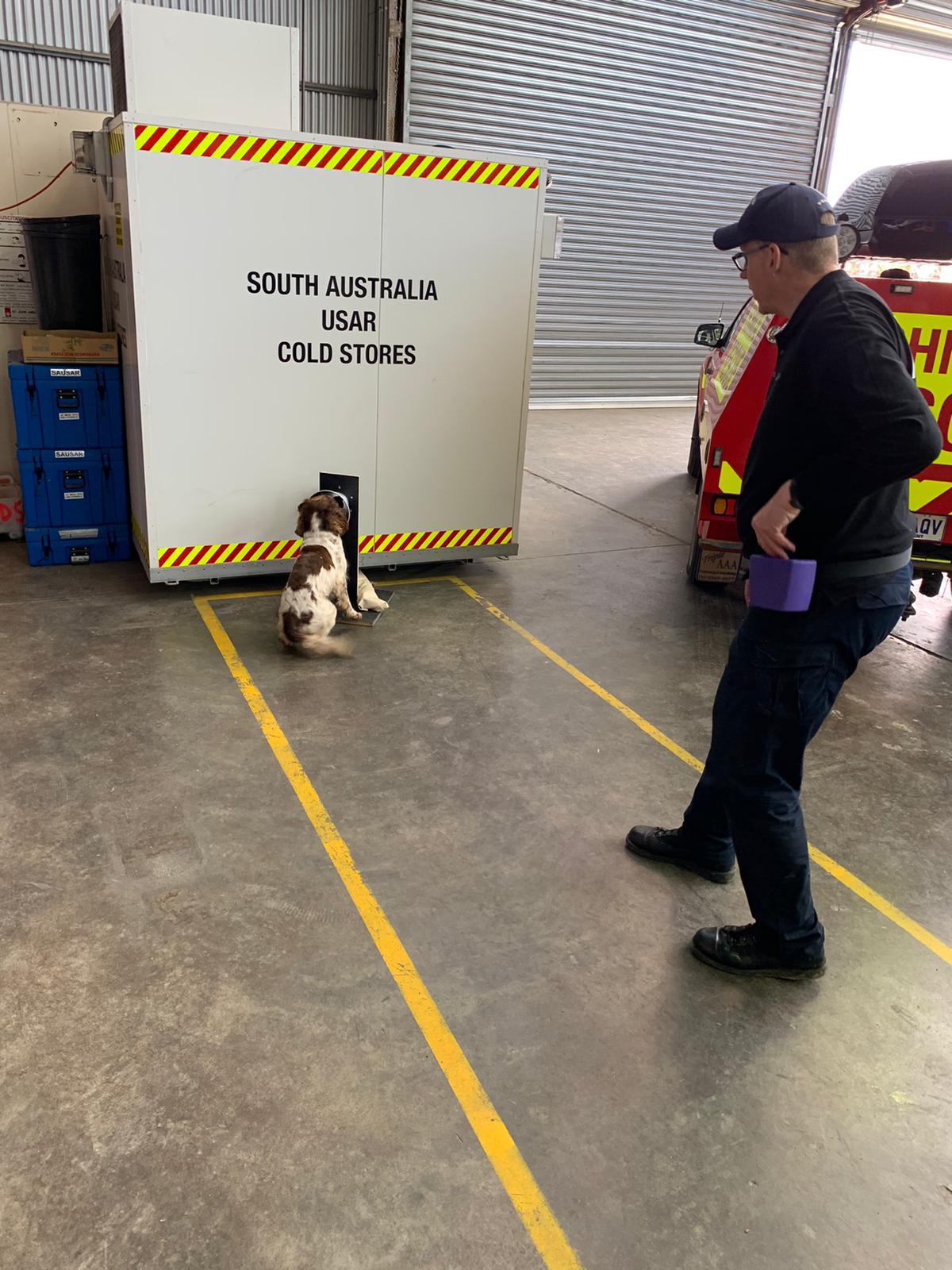


COVID Positive samples will be indicated using the following agreed alert method;

“The canine must sit in front of the hide, focusing on the hide with their nose either in the olfaction cone, partially in the cone or immediately adjacent the cone such that the canine’s body language is clearly indicating the hide containing the COVID positive (+ve) sample.”

When dogs are progressing in their training to the stage where they are to be trained on the target odour, the project ***specifically*** requires the use of hides that conform to the following specifications;

- Must be designed such that the dog cannot get direct access to / contact with the sample – preventing potential contamination of the sample or the dog.
- Must be made of metal (either stainless steel or commercial grade aluminium) that is stable and can be cleaned with ‘acetone’. The use of porous materials such PVC, HDPE or other is not supported.
- Securely incorporates a glass vessel or jar to contain the target odour sample when placed in the hide.
- Constructed in a manner that maintains suitable canine ergonomics and protection to the sample


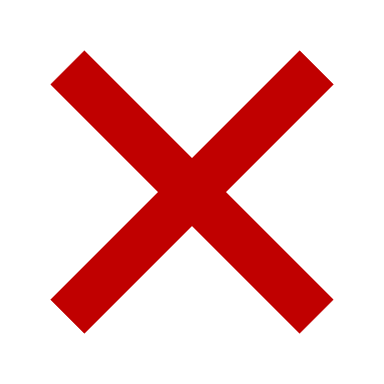

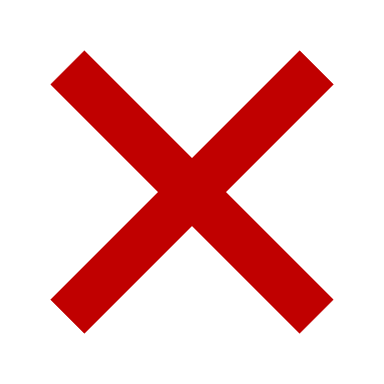

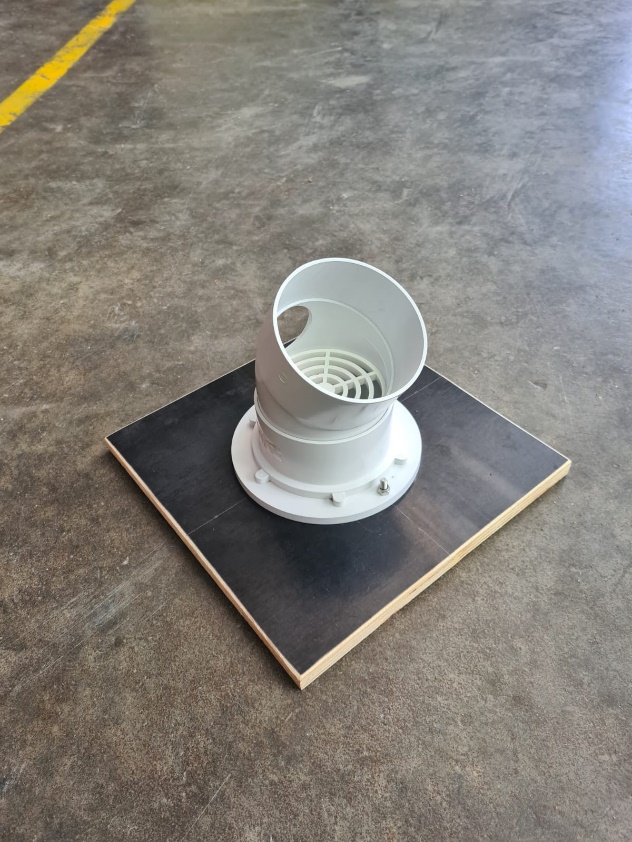

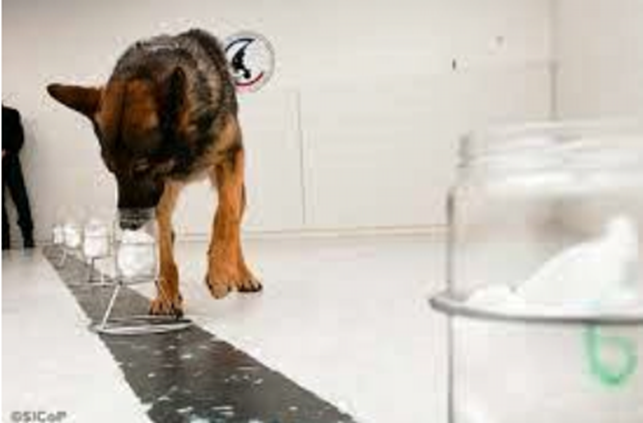


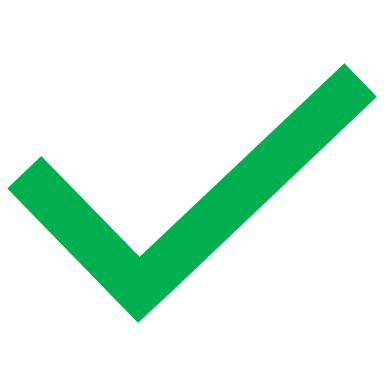

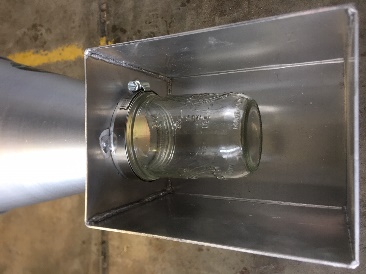

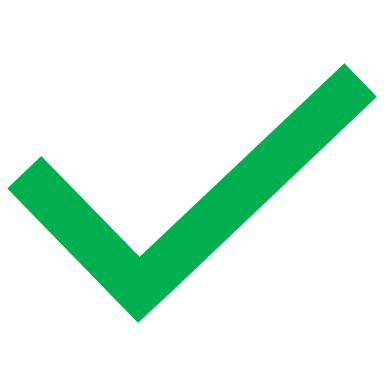

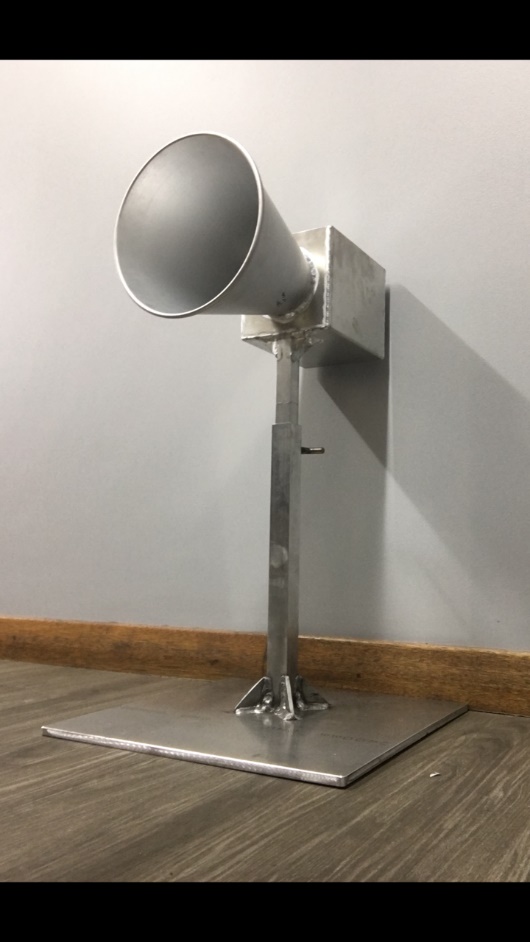

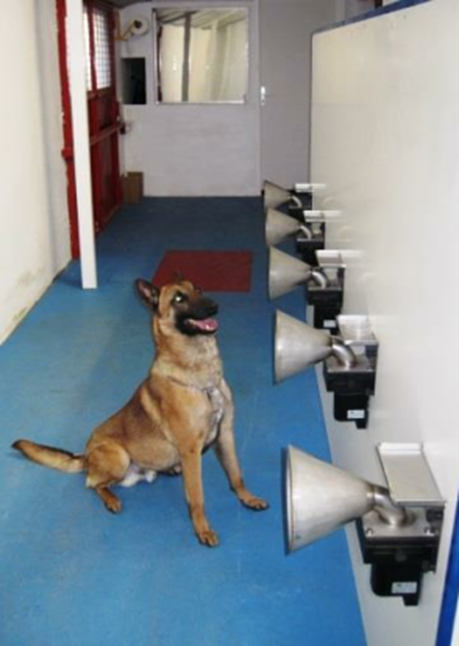


# **Section 1 – Fundamental Skills Development**

This section aims to identify a common approach for the training and development of green dogs (and dogs sourced from allied disciplines) in the core skills required to participate effectively in the study and future deployment models.

**Prerequisites:**

Only dogs with demonstrated mental (drives) and physical traits suitable for development as specific odour detector dogs should be selected for participation in this trial. Dogs should be socialised and able to work in a variety of settings / environments and free of noise and / or surface sensitivities.

Handlers / trainers should have knowledge and experience of the following concepts core concepts;

- Reward based training methods including the marker system (clicker and / or verbal markers).
- Effective food and / or prey play reward systems.
- Knowledge of behaviour shaping, targeting (‘button pressing’) and back chaining principles.
- ‘On source’ and ‘off source’ rewarding.

**Note:** For green dogs to be trained with a verbal marker and likely to be used outside of this initial project and / or handled by different handlers, a uniform verbal marker across the project is highly recommended. Verbal markers should not include words that are in common use (i.e. OK or Yes) or are difficult to deploy. The verbal marker ***‘CHIP!’*** has been shown to be easy, quick, and effective to deploy.

Key stages in the training and development of fundamental skills include;

- 1. Establishing final response alert behaviours
  2. Introduction to a training odour
  3. Introduction to scent discrimination
  4. Introduction to search skills
  5. Introduction to a blank search
  6. Validation of fundamental skills development

## **Establishing final response alert behaviours**

For the successful development of a detector dog it is common to ensure that the indication or final response alert behaviour is fully trained to the desired specification prior to a search component being added to the training regimen.

The final response alert behaviours are developed using reward-based training methods including behaviour shaping, targeting and marker system principles.

Food as a lower value reward is recommended in the ‘teaching phases’ of training to enable higher rates of repetition and to allow the dog to ‘think through’ training problems. This enables the alert behaviours to be established more effectively through the development of muscle memory.

If prey / play rewards are used the dog must have a ‘conflict free out on command’ established to ensure that a high rate of repetition can be maintained throughout the training and avoiding the introduction of stress to the teaching phase.

Play prey rewards may be tugs, tennis balls, kongs, balls on a rope, kongs on a rope or similar. Tugs and / or balls or kongs on a rope may be preferred in the operational context as they are easier to control whilst rewarding.

The use of an ‘***off source***’ reward is recommended for dogs in preparation for operational environments where it is difficult or undesirable to reward ‘on source’ (i.e. searching line ups of people, or near people not accustomed to dogs, or to avoid contamination of the area immediately around hides). On source rewarding is however used in several stages of core skills development.

Where appropriate, a training hide can be used to assist in the development of desired final response ‘alert’ behaviours. The prop should approximate the height, size and shape (ergonomics) of the hides used in the project and designed so that dogs can be easily rewarded from the training hide. During this stage in training, the hide will not be used in conjunction with the target odour, as such their construction is flexible.


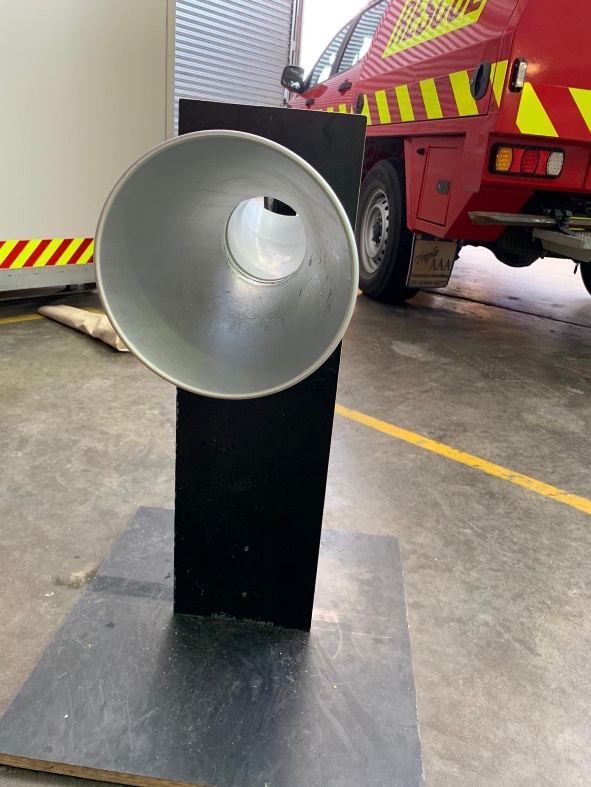

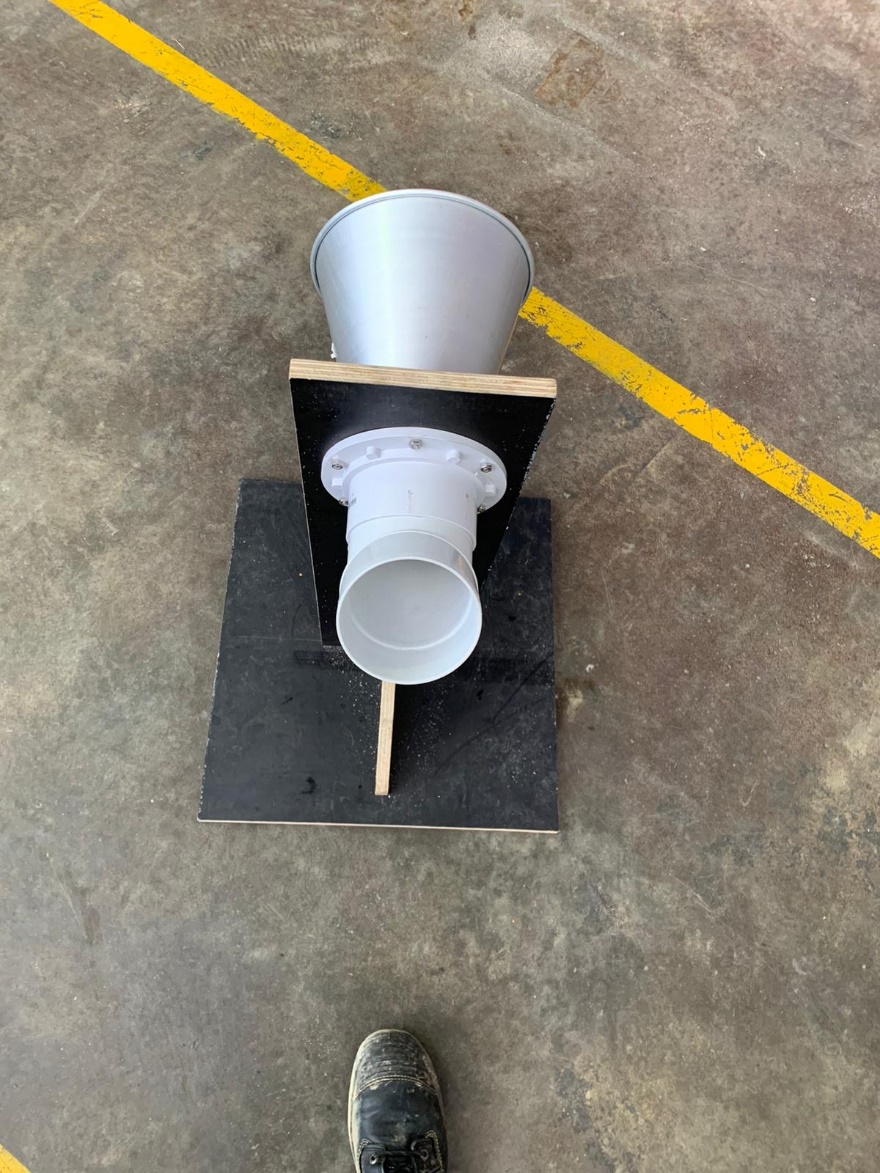

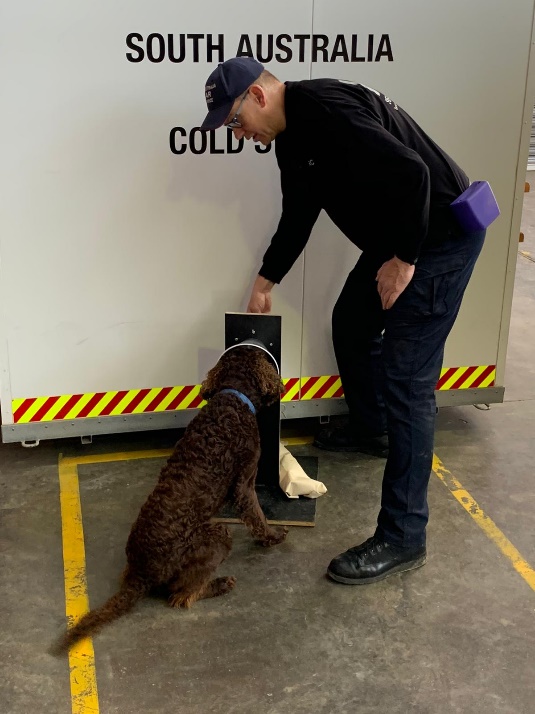


*Example of a training hide used to develop desired alert behaviours in a green or untrained dog*

**Key stages of development**

- Dog is taught the ‘value’ and ergonomics of the hide by flooding with rewards from the hide. High rates of repetition and where the dog can access a rewarding every time it places its head into the hide’s cone. Rewarding is done ‘On Source’ from the hide.
- Reward is presented but withheld, the dog pre-empts the reward coming from the hide and begins to naturally offer to put its head and nose into the cone. Dog is marked and rewarded and rewarded ‘on source’ from the hide.
- Dog consistently stays near and / or begins to target the hide and offers to put its and nose into cone without a reward initially being presented. Dog is marked and rewarded ‘off source’ by stepping away from the hide when rewarding.
- With the handler a short distance away, the dog begins to actively shuttle between the handler and the hide during repetitions. The dog is actively targeting the hide and placing nose into the hide with each repetition. Dog is marked and rewarded ‘off source’ each time it targets the hide. At this stage a search command may be introduced.
- A reward is again presented from in the hide and withheld. Dog engages the hide and is encouraged to sit. Dog is marked and rewarded from the hide each time it sits with its nose in the hide.
- A reward is presented in the hide and withheld. Dog engages the hide and naturally offers a sit with its nose in the hide ***without*** any prompting. Dog is marked and rewarded from the hide.
- With the handler near the hide the dog naturally engages the hide and actively offers a sit with nose in the hide without a reward being presented from the hide. The dog is marked and rewarded off source by stepping away from the hide as they are rewarding. A search command may be reintroduced at this stage to help indicate the start of each new repetition.
- With the handler a short distance away from the hide 3-4 meters), the dog on command actively targets the hide and offers the desired alert behaviour. Dog is marked and rewarded off source and another command to search given to signal the start of a new repetition. The use of a prey play reward systems may be introduced at this stage.
- Handler begins to add movement, changes their orientation and / or distance to the hide when giving the search command, or when the dog is targeting the hide, or when it is giving its alert behaviour. This stage begins to proof the dog against any unintentional handler cues (sign tracking) or movement when either searching and / or giving its alert behaviour. The dog is marked and rewarded off source with each repetition with preferred food and /or prey play systems.

## **Introduction to a training odour**

This stage ***should not*** be undertaken until the dog is fluent in the targeting of a hide and independently offering desired final response alert behaviours free of any handler cues (See above).

The training odour should be;

-
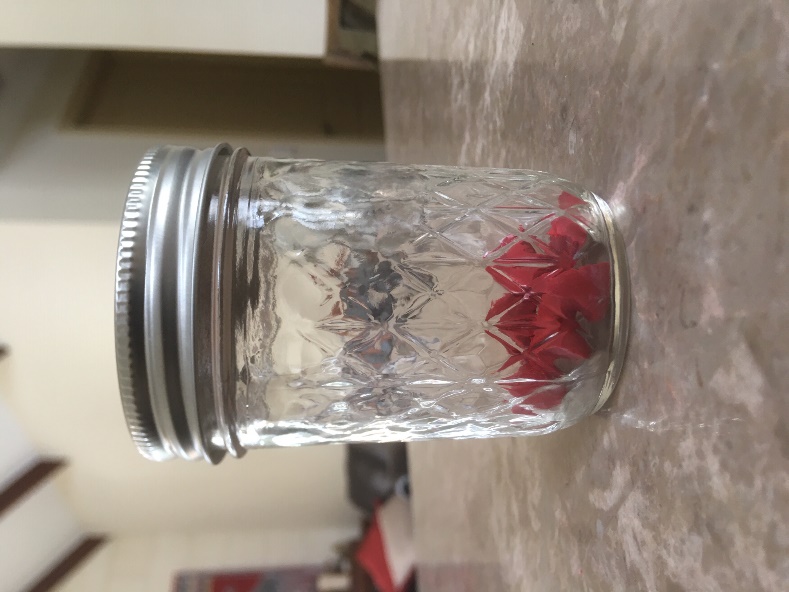
something that has relevance to the dog,
- is easily replicated and managed,
- not related to the desired target odour (i.e. no human scented objects) and
- can easily be trained out in the following stages.

The use of diced boiled ‘kong’ (see picture) or tennis ball pieces is widely considered to be suitable training odours to teach the basic principles of odour imprinting, discrimination and searching.

At this stage the training hide can be replaced with the hide used in the study (see above).

The new hide is introduced with the training odour present

**Legend for Training Diagrams:**

**1.**
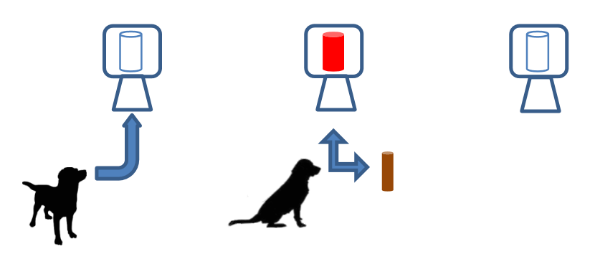
**2.****3.**

**1.** Dog searching a blank hide, or a hide containing unused medium

**2.** Dog searching a -ve sample hide

**3.** Dog indicating a hide with target odour and given a reward

**
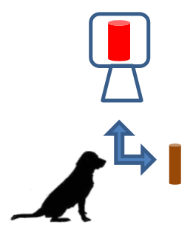
Stages of development**

- The initial acclimation is achieved by marking and rewarding every time the dog shows interest in and attempts to engage with the new hide. This stage aims to reinforce to the dog that the new chamber is valuable and targeting in the first instance is desired.
- With the handler a short distance away from the hide, the dog on command actively targets the hide and offers the desired alert behaviour. Dog is marked and rewarded off source and another command to search is used to signal the start of each new repetition. The use of a prey play reward may again be introduced at this stage.
- Handler begins to add movement, changes their orientation and / or distance to the hide when giving the search command, when the dog is targeting the hide, or when it is giving its alert behaviour. Dog is marked and rewarded off source with each repetition with the desired food and /or prey play system.

## **Introduction to scent discrimination**

For this stage, two identical hides are presented in the training space. One with the training odour and one that is blank / empty.

The hide containing the training odour is clearly marked for the handler to prevent any potential for errors during this important stage (i.e. a red dot sticker is placed on the hide containing the training odour such that the handler can see it).

An area for the dog is provided so that the dog can be placed out of view between each repetition when the hides are being moved.

**Note:** It is important to ensure throughout this stage that the dog is given every opportunity to learn through trial and error without any handler correction or cueing. The dog must learn to solve the scent problem on its own.

**Stages to development**

-
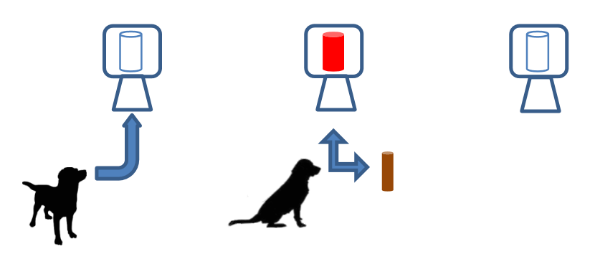
Dog is introduced to the scenario and given the command to search. The canine is allowed to investigate both hides and it is marked and rewarded when it actively investigates and targets the hide containing the training odour. ***NOTE:*** At this stage we are not asking for a final response alert behaviour but are seeking only to mark and reward the moment the dog discriminates and correctly decides to target the training odour.
- The above stage is repeated with the position of the two hides being randomly changed between repeats (with the dog out of view). The dog is marked and rewarded for making the correct decision in its odour discrimination. This stage is complete when the dog is clearly targeting the hide with training odour between the repetitions.
- On targeting the correct hide the mark and reward is withheld and the dog is begins to independently offers its final response alert behaviours on the training odour. The dog is marked and rewarded off source.
- Handler begins to add movement, changes their orientation and / or distance to the hide when giving the search command, when the dog is targeting the hide, or when it is giving its alert behaviour. Dog is marked and rewarded off source with each repetition.

## **Introduction to search**

The stage is only undertaken once the dog has clearly established the final response alert behaviours, understands a search command, and can clearly demonstrated that it can ***independently*** discriminate, target and alert on the training odour. This stage will see the number of hides increased from line ups of 2 to 4 or more hides. One hide will contain the training odour. The others will be either left blank, or may contain unused media (sterile swabs), or a distractor during later stages.

The hide containing the training odour is clearly marked for the handler. Hides containing distractors or blanks are also clearly marked for the handler.

***Note:*** During this stage it is vital that we allow the dog to learn from its own mistakes and avoid correcting or cueing a dog if it makes any wrong decision. It must learn on its own that indicating blanks and / or distractors will not result in success. We should always aim to mark and reward the correct decision to commit to odour. Failure to allow for this in training will often result in a dog that when faced with a difficult scent problem will cue off the handler (handler dependence) and potentially offer a false alert or fail to provide an alert on a target odour.

**Stages in development**

-
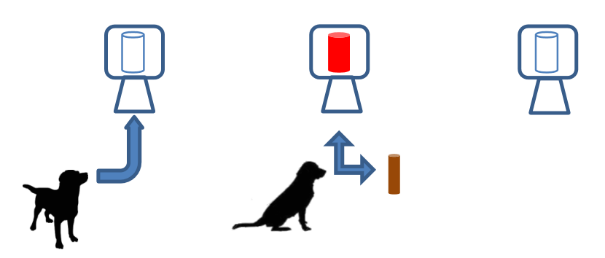
The number of blank hides is increased until the dog can actively search and indicate the training odour in line-ups of 3 - 4 or more hides. Dogs are placed out of view between each repetition and to order of the line-up changed randomly between repetitions.
- Distraction odours (i.e. food) un related to the training odour (or the study target odour) can be substituted for a blank hide. Dogs are marked and rewarded every time they ignore the blanks and distractors and currently discriminate, target and alert on the training odour.
- Handler begins to add movement, changes their orientation and / or distance to the hide when giving the search command, when the dog is targeting the hide, or when it is giving its alert behaviour. Dog is marked and rewarded off source.
- Proofing handler / environmental distractions: In addition to the above the handler and / or assistants may also add distractions such as verbals, gestures, movements, or articles (i.e. moving a chair, opening doors, making noise, another dog in a crate, reward toy in plain view etc). Should the dog become distracted the handler / assistant will become neutral offering no correction or cues and allowing the dog to refocus on its own and recommit to searching. When the dog recommits to searching and alerts correctly, the dog is marked and ***suitably*** rewarded. The level of distraction will be low at the beginning phases and increase with the proficiently of the canine. Proofing at this level will significantly reduce / eliminate the likelihood of false negative and / or false positive responses in the later stages of training, validation and deployment.

## **Introduction to a blank search**

It is important for the dog to be exposed to a blank search without any training odour present. At this stage a simple line up of up to 2-4 hides that contain no training odour is presented. Care is taken to ensure no potential for cross contamination with training odour has occurred etc.

It is important to ensure these sessions are interspersed with searches containing the training odour to ensure proficiency / accuracy.

**Stages in development**

- A simple line up of two hides is presented. On command to search the dog can investigate each hide. As soon as the dog has investigated all hides and either before it has an opportunity to present a false positive, or it begins looking elsewhere for other potential hides, it is marked and ***suitably*** rewarded.
- The process is repeated such that the dog can successful complete a blank search of up to 4 (or more) hides without offering any alert behaviours.
- The use of blank searches is then integrated into the training regimen with other searches containing the training odour. This is done to ensure that the dog can search blanks without offering a false alert.


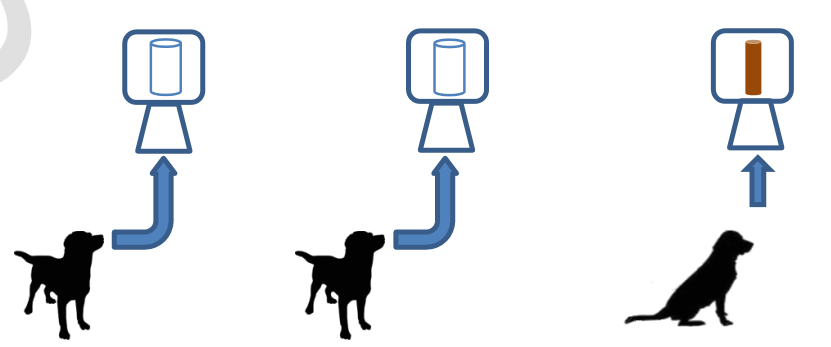

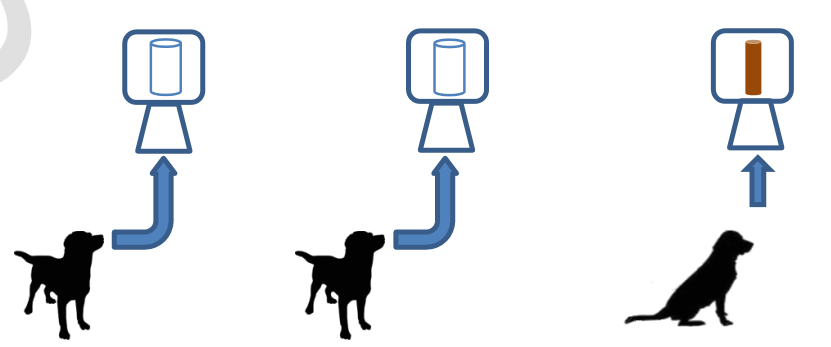

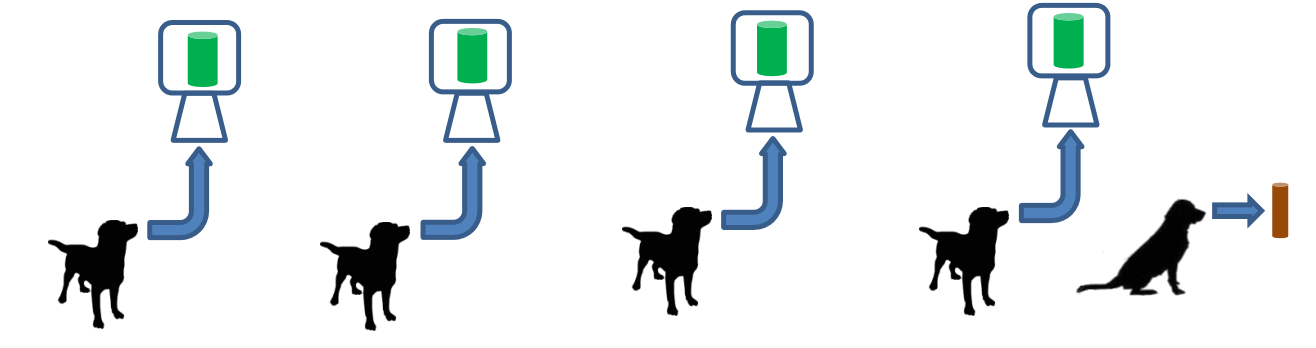


## **Validation of fundamental skills development and / or project acclimatisation**

In order to ensure that all dogs entering the COVID-19 Detector Dog project can demonstrate the development of fundamental skills a simple validation is undertaken either;

- At the end of fundamental skills development, or
- At the end of an acclimatisation period prior to commencing imprinting on COVID-19

The fundamental skills validation consists of;

- Five blind searches where each search consists of 5 hides that;
  - - contain 1 hide with target odour and 4 hides with empty jars
    - are randomly placed using a random number generator
    - are blind to the dog and handler team
- One search containing 5 blank hides. The blank search is known to the handler

All canines must complete this validation in order to demonstrate they have the necessary skills to successfully participate in the COVID-19 Imprinting phase of the project.

In order to complete the assessment, the dog must;

- systematically search hides and correctly indicate all 5 target odours presented using the agreed alert method and without giving any False Positive indications
- search all hides in the blank search without giving any False Positive indications

Results of the fundamental skills validation should be recorded using the attached training record sheet (where ‘+ve’ is used to indicate the location of the training target odour).

On successful completion of the validation, the record sheet should be signed by the project group coordinator, scanned and forwarded to the project coordinator Anne-Lise Chaber via;

- email to [anne-lise.chaber@adelaide.edu.au](mailto:anne-lise.chaber@adelaide.edu.au) , or
- mail to; ***Dr. Anne-Lise Chaber***

School of Animal & Veterinary Sciences

Leske Building  G13

Roseworthy Campus, The University of Adelaide,

SA, AUSTRALIA 5371

# **Section 2: Introduction to target odour (all dogs)**

**Requirements**

This stage will be for dogs that;

- Already have an established final response alert behaviour consistent with the study requirements
- Understand how to search a scent detection line up.

If the dog is not experience with the type of hide being used in this study, an initial acclimation phase (like that discussed in section 1) should be undertaken to acclimatise the dog to the hide. This can be done using simple behaviour shaping methods as previously discussed.

Prior to entering the imprinting phase of the project the canine must complete a fundamental skills / acclimatisation validation (See section 1.6 for further information).

During the initial stages of the introduction to COVID positive (+ve) or negative (-ve) samples, the individual samples used should be sourced from the same location to ensure any ‘background’ odour is consistent across the samples.

Hides are to be clearly marked to identify both COVID positive and negative samples as well as blank hides, or hides containing unused sterile collection media.

As a guide when training, change samples after 2 passes and try to complete a minimum 8-10 searches per dog in a training session ***(minimum 4-5 samples per dog per session)***.

Recommendations from the French studies have identified that the training regime for the detector dogs should comprise 2 sessions per day with up to approximately 2 hours taken to complete each session.

Each of the stages identified will require the team to achieve training benchmarks before progressing on to the next stage of the imprint training process.

**Notes:**

- During the initial imprint to target odour phase (1^st^ week of training with the target odour) only use fresh samples. These are samples that are to be less than 5 days old and previously unopened.
- Samples must be refrigerated when not in use and must be allowed to come up to ambient / room temperature before use in training or validation (minimum 1 hour before use)
- Always come back to the basics for dog that are slower to progress or pick up skills. Consistency at each stage in training is vital to achieving accurate and reliable results that reflect the dog’s capabilities and outcomes of other study groups.
- ***Record any indications on negative samples (false positive). PCR test may give inaccurate results. Have ALL donors of negative samples that are indicated as positive by dogs IMMEDIATELY referred for a secondary PCR test to ensure they are not asymptomatic and COVID 19 positive.***

**Data collection**

For each training session using the target odour the following data should be collected;

- Record the dog’s reactions in the record sheet
- Record the training sessions with video and download on to a designated computer.
- Establish video files for each dog / training session.
- Handers to maintain a personal training log for each dog.

**
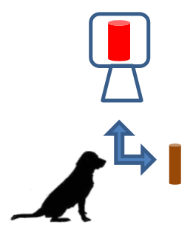
Stages in development**

**2.1 Introduction to +ve sample (target odour):** This is the same procedure is that discussed in Section 1. The hide is introduced with the +ve sample in situ. On being given the search command, the dog actively engages the hide and offers its alert behaviour. The dog is marked and rewarded, and the exercise is repeated with another command to search between each repeat. The single hide is worked until the dog is fluent in engaging the hide and offering its final response alert behaviours.

**Training Benchmark**:

Completion of a minimum of 100 searches (repetitions) using a minimum of 50 samples

**
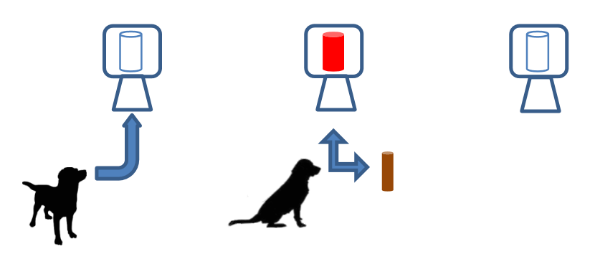
2.2 Scent discrimination with 2 hides:** (also see section 1.3). One hide with a +ve sample the other is left blank. The canine must be able to consistently search, discriminate and indicate on the hide containing the +ve sample. As training progresses +ve samples from different donors and locations may begin to be introduced into the regimen.

**Training benchmark:**

Successful completion of 10 searches using a minimum of 5 positive samples where;

- The samples have not previously been used
- The order of the hides is randomised, and
- The dog correctly indicates on all positive samples without giving any False Positive indications

**
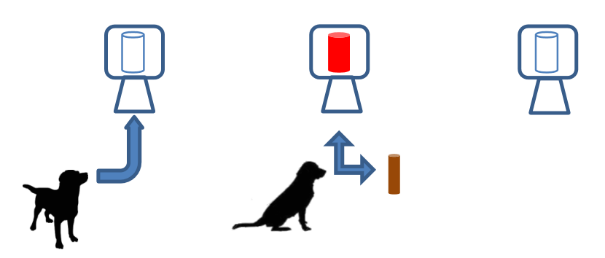
2.3 Search of up to 3 -10 hides with empty non target hides:** (also see section 1.4). One hide containing a +ve sample the remainder are left empty. The canine must consistently search a line-up that is randomised between repeats and correctly indicate the hide containing the +ve sample.

**Training Benchmark:**

Successful completion of 4 searches using 10 hides and a minimum of 2 positive samples where;

- The samples have not previously been used,
- One hide contains a +ve sample and the remaining hides are let empty
- The order of the hides is randomised, and
- The dog correctly indicates on all positive samples without giving any False Positive indications.

**2.4 Searches of up to 3-10 hides with sterile media in non-target hides**. One hide containing a +ve sample the remainder contain sterile unused collection media consistent with that used to obtain the positive sample. The canine must consistently search a line-up that is randomised between repeats and correctly indicate the hide containing the positive sample

**Training Benchmark:**

Successful completion of 4 searches using 10 hides and a minimum of 2 positive samples where;

- The samples have not previously been used,
- One hide contains a +ve sample and the remaining hides contain sterile media
- The order of the hides is randomised, and
- The dog correctly indicates on all positive samples without giving any False Positive indications.

**2.5 Introduction to the -ve sample:** As with scent discrimination training (also see section 1.3), the exercise utilises 2 hides. One with the +ve sample and the other with the -ve. The hides are clearly marked for the handler. On being given the search command the dog shall search the line-up. In the first instance the dog is initially marked and rewarded on its decision to discrimination and target the +ve sample in the presence of the -ve sample. As the dog becomes adept at detecting and targeting the +ve sample in presence of the -ve sample the dog can naturally offer its final response alert behaviours before being marked and rewarded.


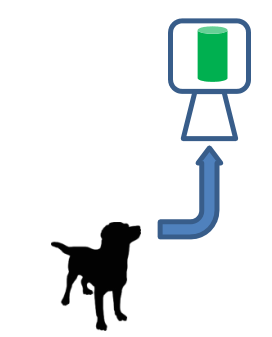

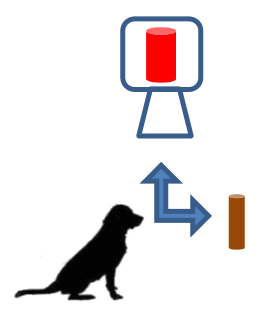


***Note:*** As in Section 1, it is important to allow the dog to learn from its own experiences and any correction or cueing due to a wrong decision is to be avoided.

***Note:*** Make sure to only introduce “real” negative for the first days of training (check medical form when selecting samples)

**Training Benchmark:**

Successful completion of 10 searches (repeats) using a minimum of 5 positive samples where;

- The samples have not previously been used
- One hide containes a +ve sample the other containes and ‘confirmed’ -ve sample
- The order of the hides is randomised, and
- The dog correctly indicates on all positive samples without giving any False Positive indications

**2.6 Searching of line ups with +ve and -ve samples:** This stage will require the dog to search line ups of up to 3 - 10 hides (also see section 1.4). One shall contain a +ve sample the remaining will contain -ve samples or hides containing sterile collection media (swabs). The hides are clearly marked, and the order of the line-up is randomised between repeats. The canine must be able to consistently search and correctly indicate the hide with the +ve sample in randomised repeats of the line-up. As training progresses, the number of hides in the line-up increases and both +ve and -ve samples from different donors are introduced to the regimen.


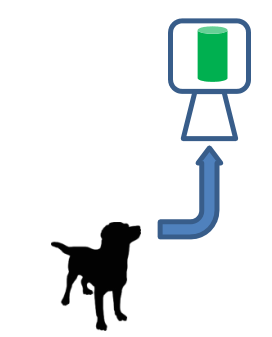

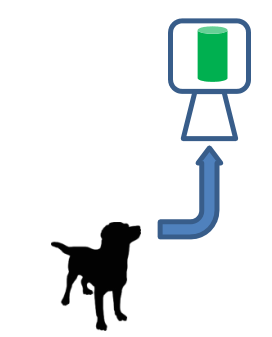

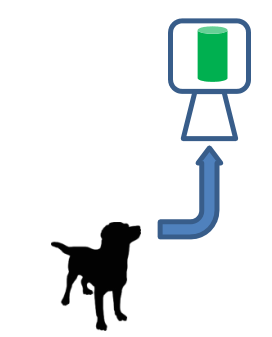

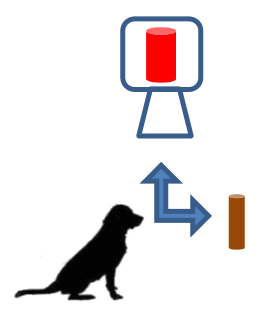


**Training Benchmark:**

Successful completion of 6 searches using 10 hides and a minimum of 3 (to a maximum of 6) positive samples where;

- The samples have not previously been used,
- One hide contains a +ve sample and the remaining hides contain -ve samples
- The order of the hides is randomised between searches, and
- The dog correctly indicates on all positive samples without giving any False Positive indications.

**2.7 Blank searches:** When the dog is consistently working line ups of 4 – 10 hides, the dog should be introduced to blank searches where all hides presented contain a -ve sample (also see section 1.5). The dog must consistently search a blank line up and not offer an indication on any of the hides. Once introduced, blank searches can be integrated into the training regimen along-side searches that contain both +ve and -ve samples.


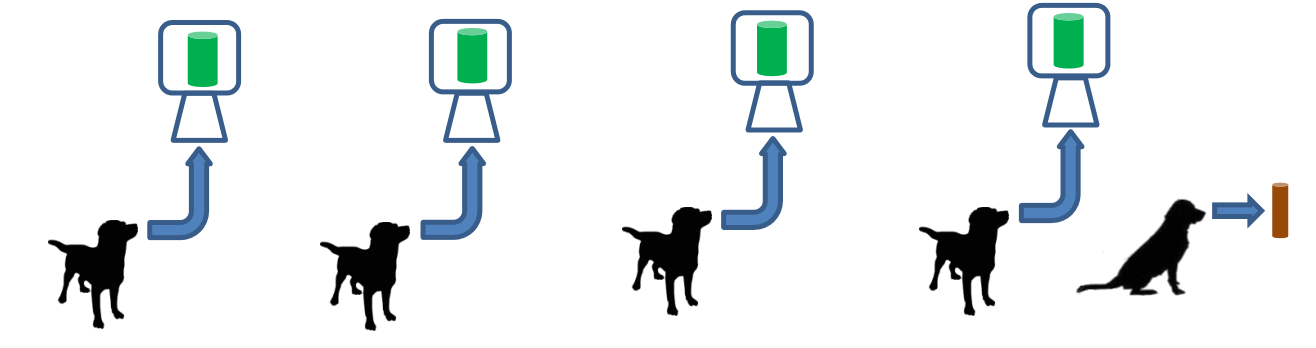


**Training Benchmark:**

The dog is able complete 2 searches containing 10 hides with -ve samples. The dog must demonstrate that it samples each hide, its body language indicates a lack of target odour and it does not give a false positive response. The line ups may be attempted separately and at any time during the training program.

**2.7 Proofing:** Handler proofing and environmental proofing (also see section 1.4) can be introduced into the scent line ups to ensure that the dog is reliably indicating +ve samples even in the presence of any handler and / or environmental distractions.

**2.8 Introduction to blind searches:** This stage involves the introduction of blind searches of between 4 – 10 hides, where the handler does not know the location of the +ve sample in the line-up. Blind searches may be introduced at various stages during the training program where searches are being conducted using sterile collection media or -ve samples.

This stage prepares the handler and the dog for the Training Validation phase which is done double blind.


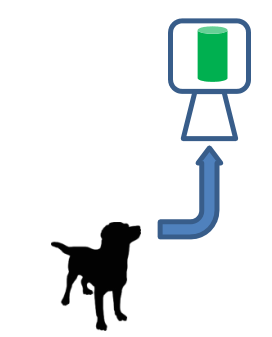

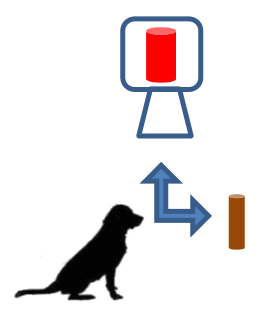

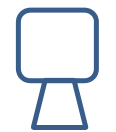


**?**


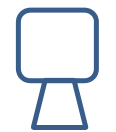


**?**


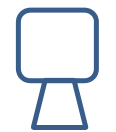


**?**


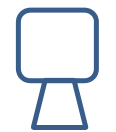


**?**


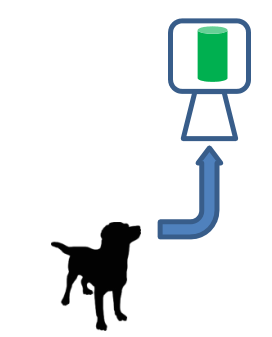

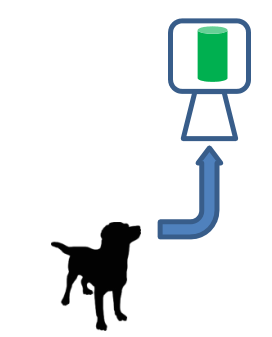


**Training Benchmark:**

Successful completion of 6 searches (repeats) using a minimum of 3 positive samples where;

- The +ve samples have not previously been used
- One hide contains a +ve sample and the remaining hides contain -ve samples
- The order of the hides is randomised and unknown to the dog and handler team, and
- The dog correctly indicates on all positive samples without giving any False Positive indications

# **Section 3: Training Validation Test**

The international COVID Detector Dog study will use a double-blind assessment for its Training Validation Test process.

**The Double-Blind Assessment (Sensitivity and Specificity)**

This assessment will consist of 10 searches (line-up), each containing a line-up of 10 hides. The layout and location of the samples in each of the 10 searches is unknown (blind) to the dog team and the study group coordinator.


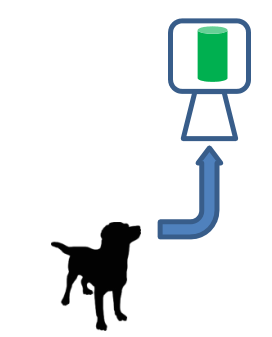

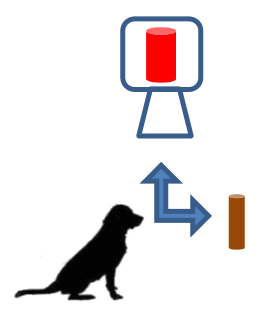

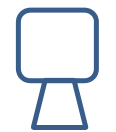


**?**


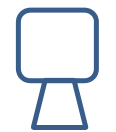


**?**


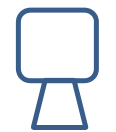


**?**


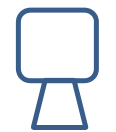


**?**


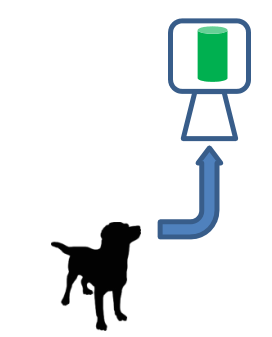

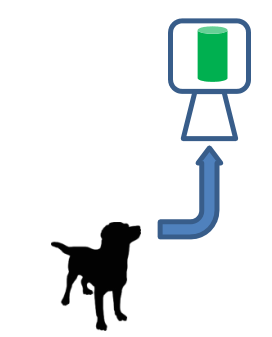


When a dog and handler team is ready for a Training Validation Test, the study group coordinator will contact the Project Manager and organise a validation pack containing 100 samples. The order and identity of the sample will be unknown to the study group assessment team and handler. Samples will be sourced from different donors and the order of each sample group of 10 (1 +ve and 9 -ve) for a search will be randomised using a random number generator. The order and identity of the samples in the validation pack is known only to the project group manager.

The dog and handler are staged in an area that is both visually and audibly separated from the testing area between each search in the assessment.

**Assessment:**

On completion of the trial the dog team will be graded as ***completed*** or ***not yet completed***. In order to successfully complete the training validation trial the dog team must score;

**Sensitivity:** Reliability in the detection of +ve samples = 90% or higher success.

**Specificity:** Reliability in only giving a positive indication in the presence of a +ve sample = 90% or higher success.

Should a dog give a ‘false positive’ indication during the Training Validation Test, the donors for the sample ***must be*** retested and subject to the outcomes of the PCR test result, the search result may be amended or omitted from the dog’s test data.

Should a dog give a false negative during the Training Validation Trial, the results will be compared with other dogs in the trial, and should any trends be identified the search result will be investigated and may be omitted from the dog’s test data.

On successful completion of the Training Validation Test the dog team will be recommended for participation in the double-blind proof of concept trial.

Should a dog be graded as ‘not yet completed’, the project coordinators in discussion with the dog teams study group manager, will identify the dog’s suitability for the project and / or any remedial training plans and a suggested date for reassessment.

# **Section 4: Guides for delivery of training sessions**

Attached are the guidelines recommended for set up of training sessions under the International COVID Detector Dog Project.

**Set Up**

Initially dogs are grouped according to size to reduce the need for adjusting the hides between dogs and ensuring maximum efficiency in the training session.

The training area should be clean and free from distractions. An adjacent space to the training area should be provided for the dog team to stage between the repeats of searches. This space should be visually (and if possible, audibly) separated for both dog and handler.

A separate staging area should be created for the storage and handling of samples. This area should have a dedicated table for samples, cleaning material, PPE, record sheets, some chairs and the storage unit for the samples.

The staging area should also have a fridge and a disposal bin for the safe collection and disposal of used PPE and cleaning products.

A video camera, plugs, charging cables and tripod / stand is to be established in the training space.

The training space will need to be large enough to include line ups of between 4 (probability 25%) - 10 hides.

Each study group should have a minimum of 5 hides up to a maximum of 10 for each training session. 10 hides will need to be provided in order to undertake the Training Validation Trial at the conclusion of the training program.

Glass jars will contain only one sample per day. We should not mix the jars to keep the scent picture intact.

Ensure a good supply of jars for the turnover on the line-ups (especially during the validation phase).

In addition to the dog and handler each session will need the following staff

- 1 observer (who will write the results)
- 1 coordinator (who organize the lines-ups – add / change the samples on the hides – decide the training order)

**PPE and Supplies**

- Masks (P2 or N95 masks)
- Nitrile (latex free) Gloves (multiple boxes sizes determined by members of study group)
- Pens
- Training record sheets
- Coloured marker pens (1 red for positive / 1 blue for negative)
- Storage boxes to contain sample jars /vials during training and transport
- Small labels (to stick to glass jars with sample code),
- Red and blue dot stickers and / or painting tape (x3 rolls) to label the hides with the sample used on the line-up during the training phase.
- Rolls of heavy-duty paper towels
- Spray bottles of filtered water and water / acetone disinfectant premix.

**Sample Storage**

- Samples must be refrigerated at approximately 4°C when not in use
- Samples must be allowed to come up to ambient room temperature before use (min 1 hour)
- Samples must be organized and stored accordingly. Make sure the labels on the sample are linked to the medical forms.
- Storage boxes should be labelled with samples to avoid confusion.
- Never touched the samples when they have been added to the lines. When transferring the samples from the container to the jars or to disposal, use forceps which are sterilised with the acetone solution after each use.
- Avoid exposing the samples to UV lights and avoid keeping the samples to open air.

**Sample Disposal**

- Used samples are to be emptied into a bin containing a dedicated labelled ‘BIO-HAZARD’ bag.
- The glass containers are to be cleaned as per the cleaning protocol described below
- At the end of each training session the used samples are sealed in the biohazard bag and then double bagged within another biohazard bag.
- The used samples are then either incinerated or Express Posted back to the project coordinator via the address below

***Dr. Anne-Lise Chaber***

School of Animal & Veterinary Sciences

Leske Building  G13

Roseworthy Campus, The University of Adelaide,

SA, AUSTRALIA 5371

**Cleaning Protocols**

The jars MUST be washed EVERY DAY (dishwasher without any powder) after the training phase.

Change gloves every time you manipulate a sample or every time you clean the supports (after each dog’s passage). You will use a very high number of gloves during the program.

Clean supports with water ONLY and paper tissue – At the end of each training day, clean your supports with a mix of acetone and water (1/3 acetone – 2/3 water).

A lot of industrial tissue rolls will be needed during the program.


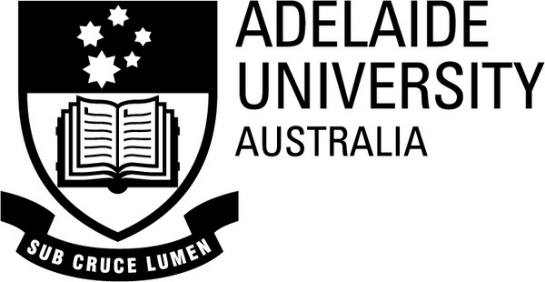


**Legend: Hides Contents**

**X** = Blank or Empty hide

**S**  = Sterile / Unused media

**+ve** = COVID positive sample

**-ve** = COVID negative sample

**D** = Enviro distractor

# **Training Record Sheet**

Name Dog:________________________________

Name Handler:_____________________________

Date:_____________ Temp:______ RH:______

| **Search**  **(Line-Up)** | **Time (24hr)** | **Sample ID No.** | **Hide Contents - Line-Up (Left to right)** | | | | | | | | | | **Observations**  **(Correct / Incorrect Indication, Other)** | **False Positive?**  **Y/N** |
| --- | --- | --- | --- | --- | --- | --- | --- | --- | --- | --- | --- | --- | --- | --- |
| 1 |  |  | 1 | 2 | 3 | 4 | 5 | 6 | 7 | 8 | 9 | 10 |  |  |
| 2 |  |  | 1 | 2 | 3 | 4 | 5 | 6 | 7 | 8 | 9 | 10 |  |  |
| 3 |  |  | 1 | 2 | 3 | 4 | 5 | 6 | 7 | 8 | 9 | 10 |  |  |
| 4 |  |  | 1 | 2 | 3 | 4 | 5 | 6 | 7 | 8 | 9 | 10 |  |  |
| 5 |  |  | 1 | 2 | 3 | 4 | 5 | 6 | 7 | 8 | 9 | 10 |  |  |
| 6 |  |  | 1 | 2 | 3 | 4 | 5 | 6 | 7 | 8 | 9 | 10 |  |  |
| 7 |  |  | 1 | 2 | 3 | 4 | 5 | 6 | 7 | 8 | 9 | 10 |  |  |
| 8 |  |  | 1 | 2 | 3 | 4 | 5 | 6 | 7 | 8 | 9 | 10 |  |  |
| 9 |  |  | 1 | 2 | 3 | 4 | 5 | 6 | 7 | 8 | 9 | 10 |  |  |
| 10 |  |  | 1 | 2 | 3 | 4 | 5 | 6 | 7 | 8 | 9 | 10 |  |  |
